# The Molluscicidal Activity of Green Synthesized Copper Oxide–Based *Annona squamosa* Seed Extract Nanoparticles on the Feeding Behavior, Biochemical, Molecular, and Immunohistochemical Alterations of *Biomphalaria alexandrina* Snails

**DOI:** 10.1007/s12011-023-03823-9

**Published:** 2023-08-30

**Authors:** Ahmed A. A. Hussein, Mona B. Abd El-latif, Marwa I. Saad El-Din, Nahla S. El-Shenawy, Olfat Hammam, Amina M. Ibrahim

**Affiliations:** 1 Medical Malacology Department, Theodor Bilharz Research Institute, Giza, Egypt; 2 Environmental Research Department, Theodor Bilharz Research Institute, Giza, Egypt; 3 Zoology Department, Faculty of Science, Suez Canal University, Ismailia, 41522 Egypt; 4 Pathology Department, Theodore Bilharz Research Institute, Giza, Egypt

**Keywords:** *Annona squamosa* L, Copper oxide nanoparticles, Characterization of CuO NPs, Molluscicidal activity, *Biomphalaria alexandrina*

## Abstract

Because of their low ecological impact, plant molluscicides have garnered much attention. The work aimed to find out if *Annona squamosa* (AS) seed extract has a molluscicidal impact on *Biomphalaria alexandrina* snails and enhances this extract by adding CuO nanoparticles (NPs). Using a scanning electron microscope (SEM), transmission electron microscope (TEM), and PANalytical X’Pert PRO X-ray diffractometer (XRD), the presence of the green *A. squamosa-based* CuO NPs (AS-CuO NPs) was confirmed. After 24 h of exposure, the half-lethal concentration (LC_50_) of AS-CuO NPs was more toxic to mature *B. alexandrina* than the aqueous extract of AS seeds (LC_50_: 119.25 mg/L vs. 169.03 mg/L). The results show that snails exposed to sublethal doses of AS-CuO NPs at LC_10_ or LC_25_ (95.4 or 106.7 mg/L, respectively) had much higher glucose levels and alkaline phosphatase activity than those not exposed. Nevertheless, there was no discernible change in the protein content in general or glycogen phosphorylase production. Histological and immunohistochemical analysis showed that snails exposed to *A. squamosa*-derived CuO NPs LC_10_ had shrinking digestive tubules and degeneration as well as vacuolation of many digestive, secretory, ova, and sperm cells, with PCNA expressing positively in the hermaphrodite gland and digestive tubule cells. The toxic profile of green CuO NPs produced by *A. squamosa* may damage the biological activity of *B. alexandrina* snails; thus, this compound could be used as a molluscicidal base. Furthermore, *B. alexandrina* proved to be a useful biomarker of nanomaterial contamination.

## Introduction

In Egypt, schistosomiasis is prevalent and is to blame for around 35% of pediatric and 70% of adult chronic liver disorders [1]. A considerable proportion of individuals involved in agricultural activities in irrigated fields are at risk of constant exposure to cercaria. This risk is particularly high during their routine activities associated with freshwater canals, which include leisure time and household activities [2]. There are four methods to stop schistosomiasis from spreading: sanitation, controlling broad intermediate host snails to stop the interaction of schistosome larval stages (miracidia, cercariae), limiting human contact to the infective stage free-swimming cercariae (reducing contacting water), and fourth, using PZQ treatments on the parasite that may induce the infection [3].

In Egypt, the freshwater snail *Biomphalaria alexandrina* serves as an intermediate host for *Schistosoma mansoni* [4]. The fact that molluscicides are selectively active, readily available, and affordable makes them essential for snail control. Other chemicals, such as copper, can kill adult snails and embryos, but they are rarely utilized in normal operations since they are absorbed by soil and organic debris [5]. It would be advantageous to investigate alternative tools and materials for managing mollusks. This approach has the potential to be cost-effective, user-friendly, and practical, presenting an appealing solution for reducing the risk of cercaria exposure during routine freshwater canal-related tasks [6]. They are also effective, exhibit water life safety, and are simple to use [7]. The use of some medicinal plants with molluscicidal properties appears to be an available and less costly alternative to chemical molluscicides [8]. The effects of plants on snails have been studied in nearly a thousand different plant types [9]. Certain natural Egyptian plants were researched for potential molluscicidal abilities [10–12]. Some plants have been discovered to be successful at controlling trematode intermediate hosts, including* Solanum xanthocarpum*, *Phytolacca dodecandra* (endod), *Thuja orientalis*,* Annona squamosa*, *Adenium arabicum*, and *Calotropis procera* [13].

The tiny, heavily branched *Annona squamosa* tree or shrub produces an edible fruit known as a sugar apple or sweetsops. The natural habitats of *Annona* species include Eastern Africa, Tropical America, and subtropical or tropical highland climates; few species, notably in East Africa and Asia, also inhabit temperate zones. It is now grown in practically all Arabian countries, including Egypt, Lebanon, Sudan, Saudi Arabia, Oman, Jordan, and Palestine [14]. The molluscicidal properties of custard apple leaves, bark, and seed were studied on *Lymnaea acuminata* snails. Seed extracts contained the highest concentration of molluscicidal activity of the plant. Acetogenins, which were extracted from the plant’s seed, had a toxic effect that was superior to that of chemical pesticides. Mixtures of custard apple seed powder, neem oil (*Azadirachta indica* A. Juss.), and cedar oil (*Cedrus deodara* Roxh) were more dangerous than the constituents of these plants alone [15]. Custard apple trash (seed) is rich in beneficial bioactive substances. Because of this, seeds have the potential to be harvested and may provide a lot of revenue for the food processing industries. Recent research suggests that a variety of plant parts left over after the first harvest, such as seeds, leaves, husks, peels, and seed coats, are a rich source of phytochemicals and nutrients and can be used to create new products for food and pharmaceutical industries [16, 17]. *A. squamosa* seed extract has hepatoprotective properties since it reduces the elevation of alanine aminotransferase (ALT), aspartate aminotransferase (AST), serum bilirubin, and alkaline phosphatase (ALP) to the normal values [18].

Nanoparticles have recently attracted much attention in various fields due to their properties such as high specific surface area and reactivity compared with bulk materials. Nowadays, among nanoparticles, metal oxides are the most common [19–21]. Copper(II) oxide is one of these transition metal oxides that are particularly interesting due to its unique properties, such as being nontoxic at low concentrations, stable, affordable, abundant, and easy preparation with numerous sizes and shapes [22, 23]. The green synthesis protocol of CuO is regarded as a suitable and ecologically acceptable alternative to the chemical method (which uses toxic chemicals as stabilizing or reducing agents) [24, 25]. Various researchers have utilized plant extracts in plant-based green synthesis protocol to fabricate CuO NPs: *Aloe vera* leaf extract [24], *Drypetes sepiaria* leaf extract [26], banana peel extract [27], *Ruellia tuberosa* leaf extract [28], and *Nerium oleander* leaf extract [29]. Moreover, CuO NPs produced by plants have been successfully applied in many areas, including cytotoxicity [30], photocatalytic protocol [26], and antibacterial activity [31].

In the present work, we evaluated the bioactivity of *A. squamosa* seed extract as a molluscicide and established an efficient technique for the biosynthesis of CuO NPs from fruit waste.

## Materials and Methods

### Chemicals

The anhydrous copper sulfate (95% analytical grade purity and 95% ethanol) was obtained from Sigma Aldrich. Distilled water was used to create extracts and metal salt solutions.

### Snails

At the Medical Malacology Laboratory of the Theodor Bilharz Research Institute (TBRI), Giza, Egypt, adult *B. alexandrina* snails (10 snails/L) were maintained and acclimated. They were housed in plastic aquaria that included 30 mg/L calcium carbonate, dechlorinated tap water (pH of 7.0 ± 0.2), and a temperature (25 ± 2 ºC) with a 12/12 photoperiod. They were also fed blue-green algae (*Nostoc muscorum*), oven-dried lettuce leaves, and TetraMin (fish food). 3 × 3 sheets of foam were used to gather egg masses [32, 33].

### Seed Extract Preparation

The fruits of *A. squamosa* L. were purchased in Giza, Egypt. The plant was recognized with the aid of Cairo’s Ministry of Agriculture in Egypt. The seeds were cleaned by removing them from the fruit, washing them with tap water, and allowing them to dry in the sun. To extract the active compounds from 50 g of seed powder, the Soxhlet equipment and 500 mL of ethanol were utilized. The powder was dried at 60 °C for 24 h in the oven after the solvent was removed using a rotary evaporator. To prepare an aqueous solution for CuO biosynthesis, the dry material was placed in a refrigerator for storage. It was filtered through the cotton bulk to get rid of any unwanted solids.

#### Biosynthesis Protocol of Green Nanoparticles

The green synthesis of CuO NPs was achieved by dissolving 0.05 mol/L of copper salt (anhydrous copper sulfate) in 400 mL of distilled water and adding 40 mL of seed aqueous extract (1.4 g of dried powder dissolved in 10 mL D.W.). The mixture was heated at 80 °C with stirring for 30 min, and the dark brown tint of the solution replaced the blue hue. Then it was centrifuged at 10,000 rpm for 10 min after 24 h of incubation to produce a pellet. The precipitate was thoroughly cleaned using distilled water and 100% ethanol, and the resultant powder was dried at 70 °C in an oven for 6 h. The powder was then subjected to a calcination process in the furnace for 4 h at 550 °C at a heating rate of 4 °C/min [9].

#### Characterization of Green-Synthesized Nanoparticles

A visible color shift was used to confirm that the CuO NP was derived from the green *A. squamosa*. After then, numerous methods were used to create the design. The solution was sonicated to avoid the particles from adhering to the copper grid, and scanning electron micrographs acquired with a JEOLJEM200CX SEM were used to analyze the morphology of the biosynthesized nanoparticles. The PANalytical X’Pert PRO X-ray diffractometer (XRD) was used to scan the dry powder between 5 and 80° (2Ɵ) at a scanning rate of 4°/min. Thermo Nicolet iS10 spectrophotometer examination using Fourier transform infrared spectroscopy (FTIR) was used to detect the composition and functional groups in charge of decreasing or stabilizing the synthesized nanoparticles. The dried nanoparticles were examined using a potassium bromide (KBr) pressed disk with wavelengths ranging from 400 to 4000/cm [34].

### Molluscicidal Activity of *A. squamosa* Seed Extract and AS-CuO NPs

#### Experiment 1: Lethal Concentration Determination

To determine the LC_90_, a series of concentrations were made from the stock solution of As-CuO NPs (150, 140, 130, 120, 110, and 100 mg/L) and of seed extract (200, 185, 170, 155, 140, and 125 mg/L) [35]. A total of 150 snails had a 24-h exposure period followed by another 24-h recovery period. Just three other snail groups of the same size were used as controls in dechlorinated water (thirty snails). In three repeats, 10 snails were used for each concentration. We tallied and looked at the observed death rates [36].

#### Experiment 2: Bioassays

In this experiment, 10 *B. alexandrina* snails (8–10 mm) were exposed to each sublethal concentration of AS-CuO NPs at LC10 (95.4 mg/L) or LC_25_ (106.7 mg/L) for 24 h, followed by an additional 24 h of recovery. Each concentration (10 snails/L) was repeated three times. For assessment, 30 healthy control snails were put up against the exposed snails.

##### Feeding Behavior

After being exposed to sublethal levels of AS-CuO NPs for 24 h, a subsequent 24-h period of recovery was allowed before proceeding with the evaluation process. The following morning, twelve snails from each group received one disk of lettuce (about 180 mm^2^). The next morning, we gathered the last bits of lettuce disk for every snail and digitally scanned them [37]. The surface area of the remaining food was determined using ImageJ to analyze the photographs [38]. The amount of lettuce consumed by each snail was then calculated by subtracting the surface area of the measured lettuce that was left over from the total lettuce that was offered at the beginning.

##### Tissue Preparation for Biochemical Assays

The glass Dounce homogenizer was used to crush and homogenize the soft bodies of the snails from the control and exposed groups (1 g tissue/10 mL phosphate buffer). For the biochemical test, centrifugations were employed to obtain supernatants (at 3000 rpm for 10 min at 4 °C). According to the attached brochure in the Biodiagnostic kits, alkaline phosphatase and phosphorylase activity, glucose, and glycogen concentrations, and total protein were all assessed (Biodiagnostic Dokki, Giza, Egypt). The Doumas technique was used to calculate the total protein.

##### Histopathological and Immunohistochemical Studies

Mature *B. alexandrina* snails (8–10 mm) were given a 24-h exposure to AS-CuO NPs. According to [39], the hermaphrodite and digestive glands were removed and processed. The glands were fixed in Bouin’s solution, embedded in paraffin wax, sectioned (4-µm thick), and stained with hematoxylin and eosin [40]. The micrometer-thick sections were examined under a Zeiss microscope (Carl Zeiss Microscopy GmbH 07,745 Jena, Germany).

Immunohistochemical examinations of proliferating cell nuclear antigen (PCNA) were performed on snail sections cuts from the paraffin blocks with commercially available anti-mouse PCNA antibodies (Santa Cruz Biotechnology, CA, USA) at the optimal working dilution of 1:100 according to [41]. The percentage of brown that is stained nuclear (PCNA) was examined in 10 microscopic fields under Zeiss light microscopy at × 200. The percentage of brown stained nuclei (PCNA) was quantified under the light microscope with a magnification of × 40 (B 40, Olympus, Japan). For each section, 10 microscopic fields were arbitrarily selected for investigation and were counted in 25 squares of ocular micrometer and the quantity of the brown stained nuclei in the selected area was determined [42].

##### Comet Assay (Rapid Genotoxicity Assessment)

To detect DNA single-strand breaks, [43] used the SCGE/comet assay on the head foot of treated and controlled snails. They produced a combination of 0.5% Low Melting Point Agarose (LMPA) and 1.0% Normal Melting Point Agarose (NMA) and heated it to almost boiling point. The slide should be placed on a tray to dry. The head foot was placed in 1-mL cold HBSS containing 20-mM EDTA/10% DMSO and minced into fine pieces. In total, 5–10 L was extracted and blended with 75 L LMPA. Microgel slides containing treated cells were electrophoresed under alkaline conditions, followed by staining with 80 L 1X Ethidium Bromide under yellow/dimmed light. EtBr-stained DNA was visualized using a fluorescence microscope with a 40 × objective. Image analysis was carried out using Kinetic Imaging, Ltd.’s Komet 5 software attached to a CCD camera. It defines regions of interest (ROIs) for the comet head and tail, measures fluorescence intensity in these ROIs, and calculates tailed % and untailed %. It also measures comet tail length and total comet length to determine tail length % and measures fluorescence in the comet tail region and the total cell for tail DNA%. The tail moment is obtained by multiplying tail length% and tail DNA % in 80 to 100 randomly chosen cells per sample. Statistical analysis of the data was conducted to compare the levels of DNA damage between experimental groups.

### Statistical Analysis

Probit analysis using SPSS version 20 was used to determine lethal concentration values. GraphPad Prism version 8 was used for data analysis, which included a one-way analysis of variance (ANOVA) for comparing the means of experimental and control groups after validating the normality of the data using the Shapiro–Wilk test.

## Results and Discussion

### Characterization of Green AS-CuO Nanoparticles

**Fig. 1:**
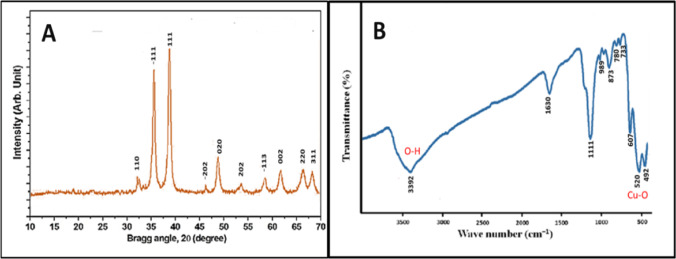
XRD (**A**) and FTIR (**B**) patterns of the biosynthesized *A. squamosa* CuO nanoparticles

*A. squamosa* oxidized copper nanoparticles were produced using a green approach and studied using XRD, FTIR, SEM, and TEM. The XRD diffraction results (Fig. 1A) revealed a pattern that corresponded to CuO data from the Joint Committee on Powder Diffraction Standards (JCPDS). Figure 1 illustrates the XRD spectrum of *A. squamosa* AS-CuO NP, which appeared monoclinic (JCPDS 72#629). The diffraction peaks at 2 Ɵ = 32.521, 35.554, 38.731, 48.736, 53.413, 58.316, 61.571, 65.808, 68.142, and 72.416 0 were assigned to the planes (110), (− 111), (111), (− 202), (020), (202), (-113), (002), (220), and (311), respectively [44, 45].

The production of CuO was shown by the presence of peaks at 2 thetas = 35—39 0, which are the principal primary diffraction peaks of the − 111,002 and 111,200 planes [31]. Also, the diffraction peaks were crisp and distinct, indicating a well-crystalline phase [46]. Furthermore, the absence of additional impurity peaks (such as Cu (OH)_2_ or Cu_2_O) indicates the purity of the green synthesized CuO NPs [47]. Figure 1B presents the FTIR spectrum of AS-CuO NP. The absorption band 3392/cm validated the stretching and vibrating frequency of the hydroxyl (–OH) group of absorbed H_2_O molecules on the surface of CuO NPs [48]. The amide I bond was assigned the band at 1630/cm. The bands at 1010 and 1111/cm were caused by phenolic and carboxylic groups, respectively [49].

**Fig. 2:**
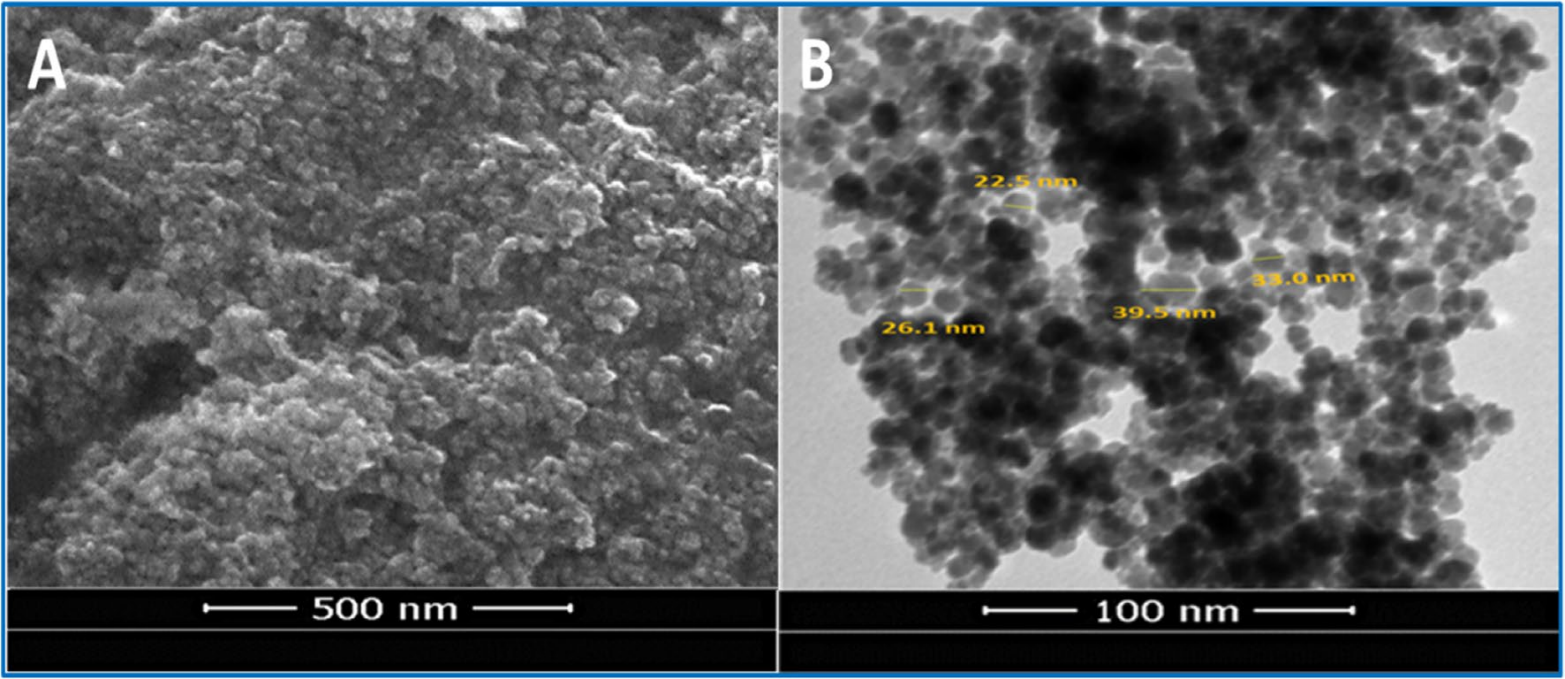
SEM (**A**) and TEM (**B**) micrographs of the biosynthesized *A. squamosa* CuO nanoparticles

Moreover, strong peaks at 598, 520, and 492/cm were assigned to Cu–O vibrations, confirming the biosynthesis of green CuO nanoparticles [30]. It was believed that the bands under 1000/cm that connected to metal–oxygen bonds would construct the AS-CuO NPs properly [30]. No absorption peaks for Cu_2_O were indicated. Moreover, Cu_2_O absorption peaks between 605 and 660/cm were not found, confirming the excellent purity of the produced CuO nanoparticles [50]. SEM and TEM images illustrate the morphological properties of green-produced AS-CuO NPs. The SEM picture identified the morphological form and structure of the produced chemical. In the current study, the produced CuO nanoparticles from *A. squamosa* seed extract had a semi-globular small-shaped appearance within the nanometric scale, as shown in Fig. 2A, and the particles looked to be agglomerated [51].

To complete the morphological report, the shape and size of the produced CuO nanoparticles were recorded using a TEM image, as shown in Fig. 2B. The TEM micrograph, like the SEM photo, showed CuO nanoparticles with semispherical structural shapes [52], and the particle’s average size was 30.27 nm, with white and black sections indicating hydrophobic and hydrophilic nature [53]. The aggregation of nanoparticles may be caused by biological components in the synthesis process [54] or by the high surface energy of the generated CuO nanoparticles as a result of the manufacturing procedure in an aqueous medium [55, 56].

### Molluscicidal Activity

From the calculated half-lethal concentration and after 24 h of exposure, AS-CuO NPs were proven to work more effectively than the aqueous extract of *A. squamosa* against adult *B. alexandrina* snails (LC_50_: 119.25 and 169.03 mg/L, respectively) (Table 1). The LC_50_ value must be determined since it assists in calculating the safe amount or tolerance threshold of any contaminants [57]. [58] studied the impact of CuO NP bioaccumulation in the freshwater gastropod *Potamopyrgus antipodarum* snail and discovered substantial reductions in survival, growth, and reproductive rate exposure to the concentration of 207 μg of Cu per g dry weight sediment for 14 days.

**Table 1:** Molluscicidal activity of AS-CuO NPs and *A. squamosa* aqueous seed extract after 24 h of exposure to *B. alexandrina* snails

Concentration (mg/L)	LC_10_	LC_25_	LC_50_	95% confidence limits	LC_90_	Slope
AS-CuO NPs	95.40	106.70	119.25	106.50–129.10	143.03	1.10
*A. squamosa* seed extract	146.30	157.09	169.03	116.70–188.19	191.72	1.11

**Fig. 3:**
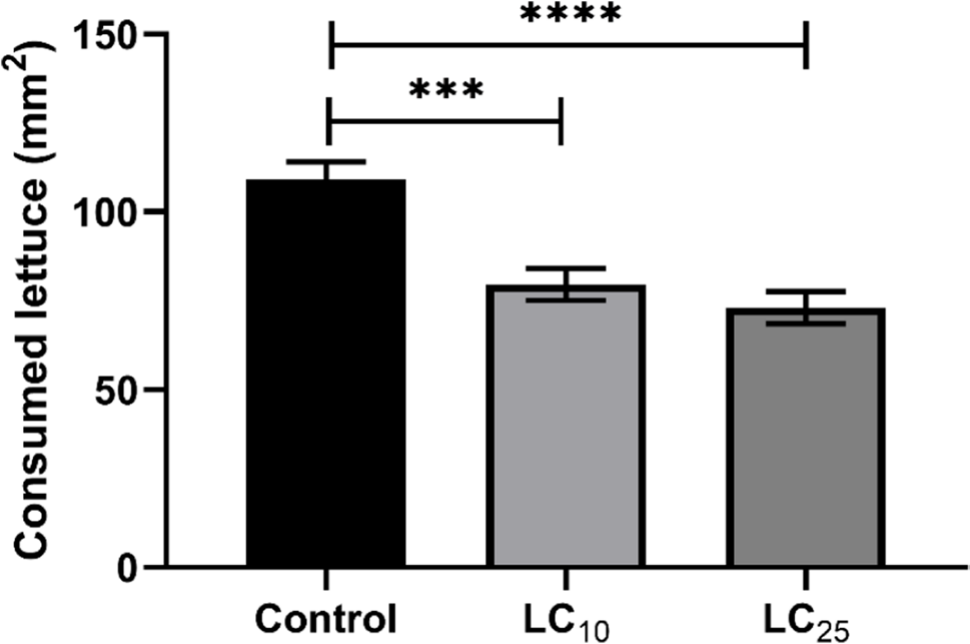
The effect of the sublethal concentrations LC_10_ or LC_25_ (95.4 or 106.7 mg/L, respectively) of AS-CuO NP exposure on the feeding behavior of *B. alexandrina* snails. ***, **** = highly significant compared to control at *P* < 0.001; 0.0001, respectively

The feeding behavior of *B. alexandrina* snails was altered after exposure to the sublethal dosages LC_10_ or LC_25_ (95.4 or 106.7 mg/L, respectively) of AS-CuONPs (Fig. 3). *B. alexandrina* snails subjected to the sublethal doses LC10 or LC25 of AS-CuO NPs showed significantly reduced eating behavior, and this decrease was concentration-dependent. One explanation might be the toxic effect on these snails’ digestive glands, which is responsible for food digestion and absorption, which was confirmed in this study by the presence of histological damage appeared in the tissue sections.

The findings also showed that after the snails were exposed to sublethal concentrations of AS-CuO NPs at LC_10_ or LC_25_ (95.4 or 106.7 mg/L, respectively), ALP activity and glucose concentrations were markedly higher in the treated groups than those of the control one. One explanation of glucose results may be linked to the results of immunohistostaining of PCNA as will be explained later. The extract’s toxicity could lead to an enhanced cell turnover and increased cell death in the digestive gland, triggering a compensatory response that upregulates PCNA expression as a marker of increased cell proliferation. This enhanced cell proliferation may be linked to accelerated glycolysis as cells may be actively utilizing glucose for energy during increased proliferation.

**Fig. 4:**
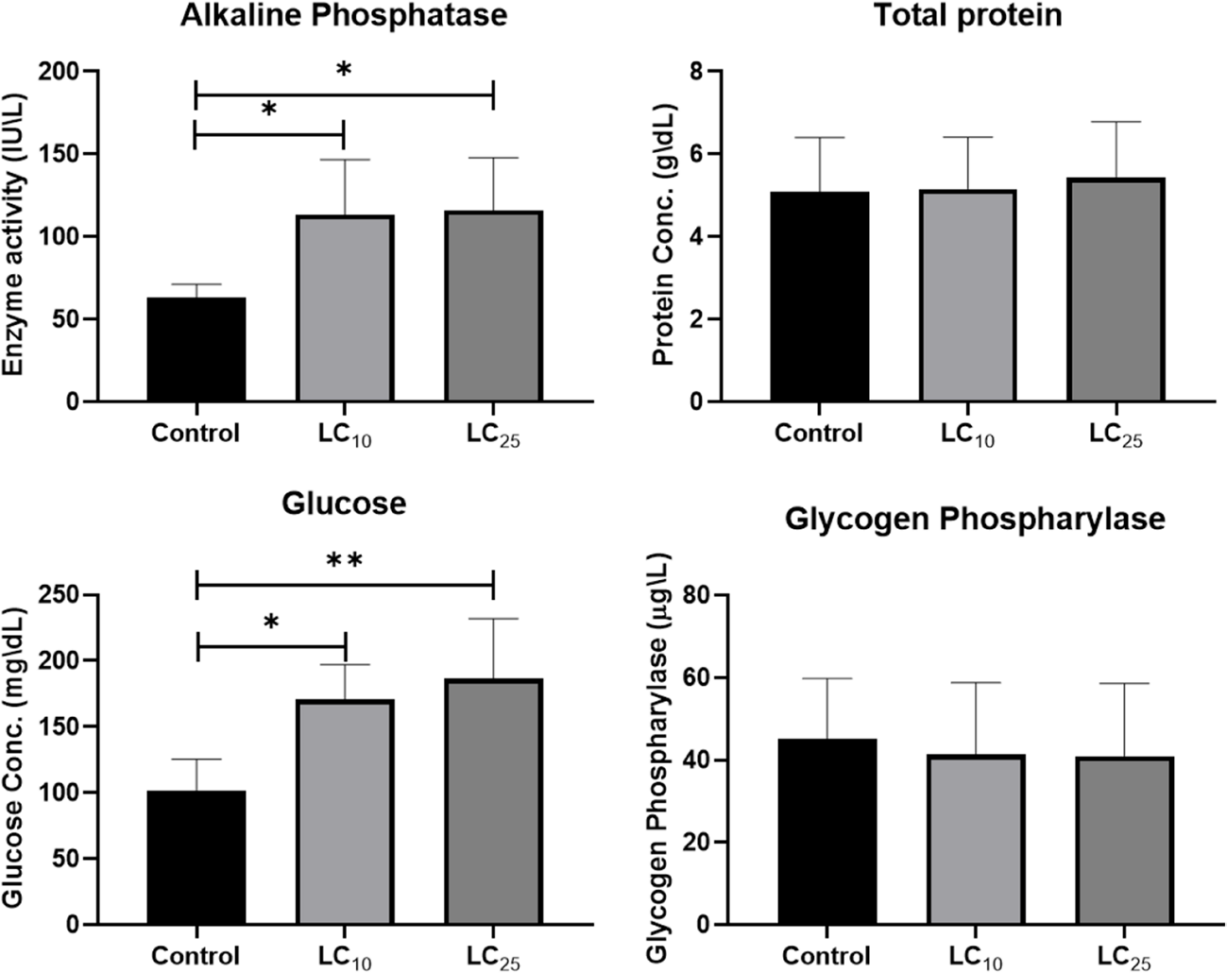
The effect of AS-CuO NP exposure on alkaline phosphatase, total protein, glucose, and glycogen phosphorylase of *B. alexandrina* snails. * = significant compared to control at *P* < 0.05. ** = highly significant compared to control at *P* < 0.01

However, neither the total protein content nor the glycogen phosphorylase activity was significantly affected by AS-CuO NPs compared with the control group (Fig. 4). The latter might be explained by the requirement of a long-term condition to modify glycogen and total protein levels, a circumstance that was not existent during this study [59]. Another explanation would be that the animals try to go back to normal following the NPs’ detrimental impact on protein production [60, 61]. From another perspective, the energy sources used by the snails and the AS-CuO NPs’ LC_10_ of the examined plant considerably reduced but not statistically different from the soft tissue’s glycogen content while raising the hemolymph’s glucose level. The activity of the active component of the plants under investigation, which inhibits snails from ingesting oxygen and instead initiates anaerobic respiration, may clarify this. The snail must accelerate glycolysis to refuel its energy requirements, which lowers the amount of glycogen present and raises the level of glucose in the hemolymph. This conclusion comes with the findings of tests using methanol extracts of *Euphorbia pseudocactus*, *Yucca* *aloifolia*, and *Portulaca oleracea* [62].

The effects of other NPs also showed a similar pattern. For instance, the *Biomphalaria glabrata* snails’ capacity for reproduction and survival was adversely affected by AS NPs [63]. When *B. alexandrina* snails were exposed to the sublethal concentrations LC_10_ or LC_25_ of AS-CuO NPs, the activity of alkaline phosphatase and the levels of glucose were significantly higher than that in the control group. This lack of difference between the two treated groups in ALP activity indicates that the impact of the treatment on this enzyme activity may have reached a plateau or saturation point at LC_10_, and increasing the treatment concentration to LC_25_ did not lead to further changes in alkaline phosphatase levels. These effects were mirrored in the biochemical and histological parameters.

**Fig. 5:**
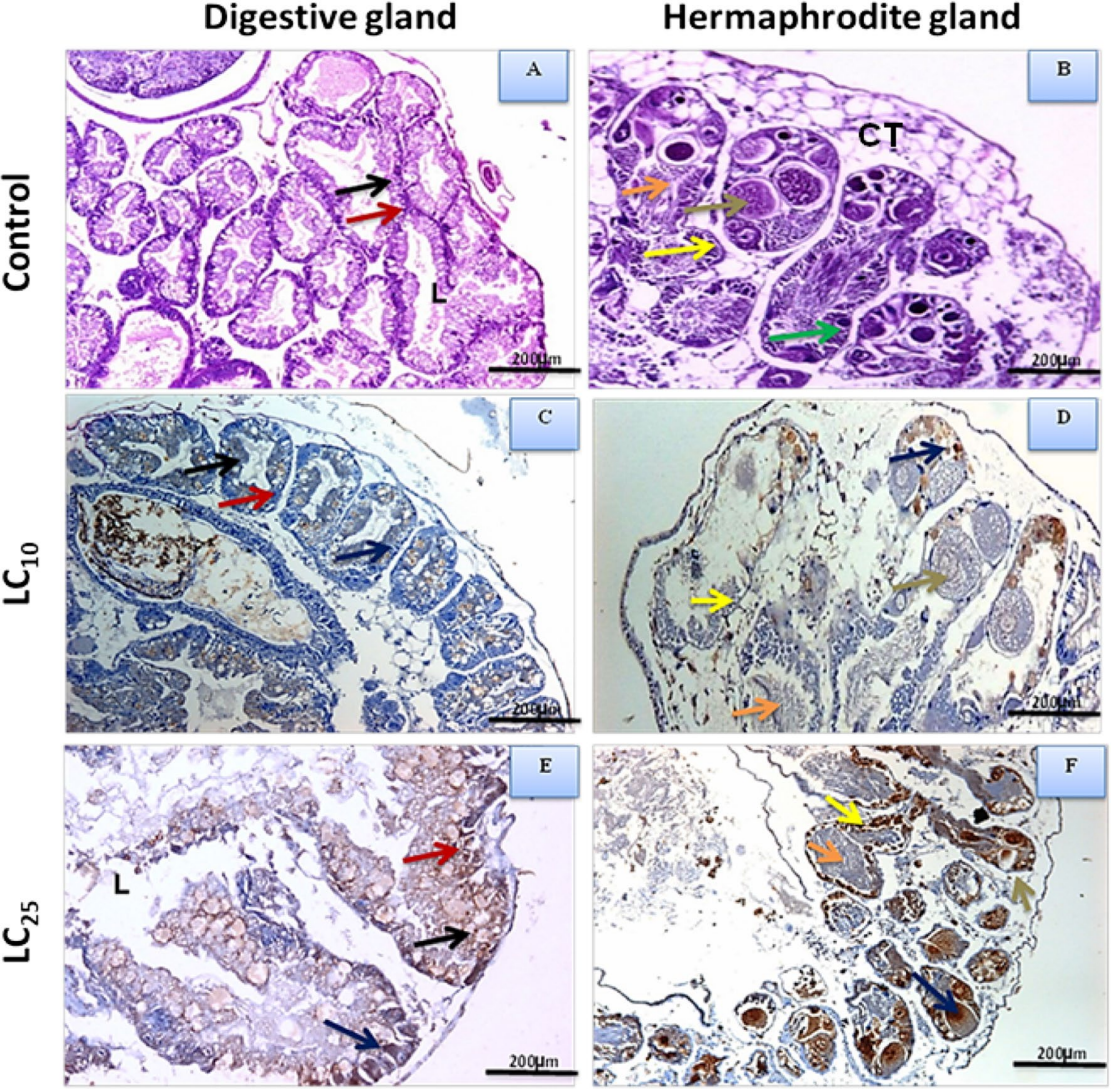
Light micrographs of* B. alexandrina* snails, showing **A** digestive and **B** hermaphrodite glands of normal snails were no expression of PCNA (immunohistochemistry for PCNA, × 200). Digestive cells (black arrow), secretory cells (red arrow); lumen (L); CT, connective tissue; mature ovum (brown arrow); oocytes (green arrow); sperms (orange arrow); and spermatocytes (yellow arrow). **C** The digestive and **D** hermaphrodite glands of exposed snails to AS-CuO NPs LC_10_ showing expression of positive expression of PCNA in the cells of both digestive and hermaphrodite glands (25%) (blue arrow) (immunohistochemistry for PCNA, × 200). **E** Digestive and **F** hermaphrodite glands of exposed snails to AS-CuO NPs LC_25_ showing increased expression of PCNA in the interstitial cells (85%) (blue arrow) (immunohistochemistry for PCNA, × 200). Degeneration in digestive cells (black arrow), secretory cells (red arrow); lumen (L). Degenerated mature ovum (brown arrow); sperms (orange arrow), and spermatocytes (yellow arrow)

The normal digestive gland of *B. alexandrina* snails was histologically shown to have a large number of tubules. A digesting and secretory cell lining covers each tubule (Fig. 5A). Furthermore, the control group’s hermaphrodite gland consists of several acini, with the female acini having mature oocytes in the follicular center and numerous peripheral oocytes and the male acini having spermatozoa in the center and spermatocytes on the acinus wall (Fig. 5B). When *B. alexandrina* snails were treated with *A. squamosa*-based CuO NPs LC_10_, the digestive tubules shrank, along with numerous digestive, secretory cells, ova, and sperms, and PCNA was positively expressed in the cells of both the digestive (Fig. 5C) and hermaphrodite glands (Fig. 5D) (25%). These changes may lead to less frequent eating. PCNA, a biomarker for hepatotoxicity, was utilized to validate these abnormalities by immunohistochemistry [64]. When *B. alexandrina* snails were exposed to LC_25_ AS-CuO NPs, they developed degenerations in the digestive, secretory, sperm, and ova, with increased expression of PCNA (85%) in the digestive tubules (Fig. 5E) and the hermaphrodite gland (Fig. 5F). In the control group, however, there was no expression of PCNA in either gland, but it was present in 25% and 85% of the cells following exposure to LC_10_ or LC_25_, respectively. This method uses the interaction between antibodies and antigens to determine whether any antigens are present in the tissue sections [65]. This damage might be caused by the snails’ toxic biological system as a result of exposure to nanoparticles [66]. This result is similar to that of a study on the marine mollusk *Mytilus* sp., in which PCNA was assessed by qPCR and shown to be increased following exposure to nanoplastics [67]. Furthermore, the current findings agreed with those of [57], who found that PCNA was positively expressed, after the LC_25_ treatment of the methanolic extract of *N. oleander*, in the ova, sperms, and digestive glands. The histological examination of the digestive gland in the current study strongly supports this concept, which is also consistent with findings from earlier investigations [61, 68].

**Fig. 6:**
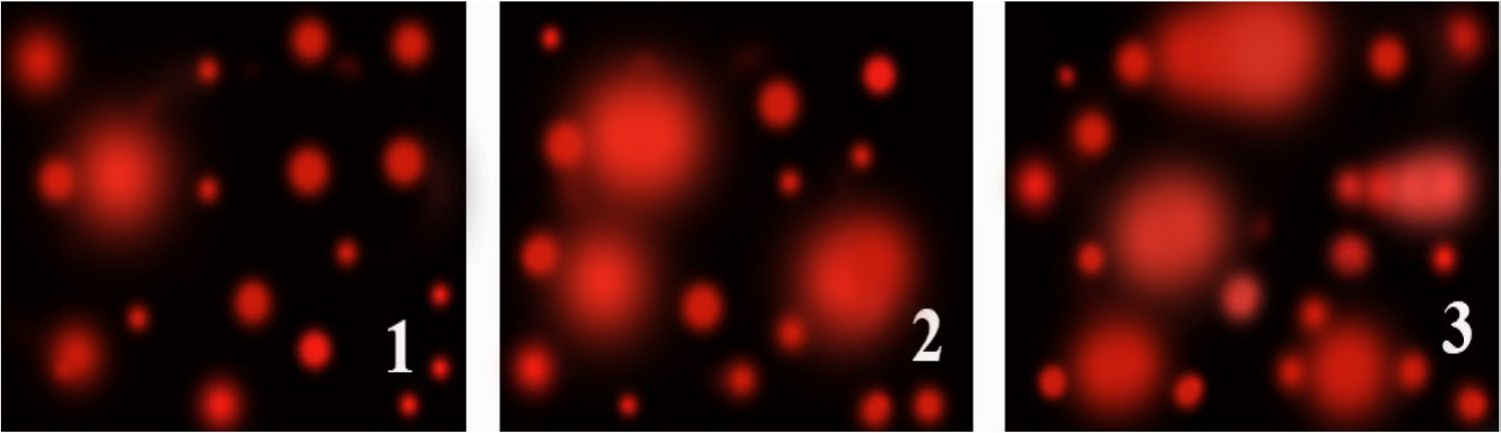
The effect of AS-CuO NP exposure on DNA of *B. alexandrina* snails by alkaline comet analysis. **1** Control *B. alexandrina* snails. **2** After exposure to the sublethal concentrations LC_10_ 95.4 mg/L of AS-CuO NPs. **3** After exposure to the sublethal concentrations LC_25_ 106.7 mg/L of AS-CuO NPs

Current results about genotoxicity revealed that control snails subjected to sublethal dosages of AS-CuONPs LC_10_ or LC_25_ (95.4 or 106.7 mg/L, respectively) had markedly reduced percentages of tailed DNA, DNA in the tail, tail length, and tail moment (Fig. 6 and Table 2). The SCGE/Comet test was used to identify single-strand breaks in DNA (Ibrahim and Ghoname, 2018). Selenium nanoparticles caused DNA damage in *B. alexandrina* snails, which increased the percentage of the comet, tail length, DNA in the tail, and tail moment after exposure to sublethal concentrations than control snails [69]. These findings agree strongly with their findings. As pointed out by [70], DNA damage may result in the production of oxidative stress.

**Table 2:** Effect of AS-CuO NP exposure on DNA of *B. alexandrina* snails by alkaline comet analysis

Groups	Tailed %	Untailed %	Tail length %	Tail DNA %	Tail moment
Control	3	97	1.41 ± 0.09	1.53	2.16 ± 0.11
LC_10_	9	91	2.49 ± 0.10*	2.77	6.90 ± 0.09*
LC_25_	15	85	3.11 ± 0.08*	2.95	9.17 ± 0.1**

Data represent mean ± SD. * = significant compared to control at *P* < 0.05. ** = highly significant compared to control at *P* < 0.01

In conclusion, the study revealed the harmful impact of green synthesized CuO NPs made from *A. squamosa* seed extract (AS-CuO NPs) on *B. alexandrina* snails and therefore might be employed as a molluscicidal agent. Moreover, *B. alexandrina* snails might be employed as a bioindicator of nanomaterial contamination.

## Data Availability

The datasets generated during and/or analyzed during the current study are available from the corresponding author on request.
